# Efgartigimod as adjunctive therapy for immunotherapy-dependent autoimmune diencephalitis: A case report

**DOI:** 10.1097/MD.0000000000050637

**Published:** 2026-09-11

**Authors:** Chang Li, Xiang Yin, Menghan Jia, Jie Shao, Li Cui

**Affiliations:** a Department of Neurology, The First Hospital of Jilin University, Changchun, China.

**Keywords:** autoimmune diencephalitis, case report, efgartigimod

## Abstract

**Rationale:**

Autoimmune diencephalitis is a rare neurological disorder. Our hospital recently admitted a case of immunotherapy-dependent autoimmune diencephalitis. The patient showed significant improvement after adjunctive treatment with efgartigimod, providing new insights into the treatment of autoimmune diencephalitis.

**Patient concerns:**

The patient, a middle-aged male, presented with acute onset of symptoms lasting more than 3 months, including intermittent headache, fever, epileptic seizures, and impaired consciousness.

**Diagnoses:**

Laboratory tests and magnetic resonance imaging confirmed autoimmune diencephalitis.

**Interventions:**

The patient was treated with glucocorticoids, intravenous immunoglobulin, mycophenolate mofetil, and efgartigimod.

**Outcomes:**

After the addition of efgartigimod, glucocorticoid tapering was successfully achieved, and the patient gradually recovered.

**Lessons:**

This case demonstrates the favorable effect of efgartigimod as an adjunctive therapy for autoimmune diencephalitis, effectively controlling symptoms and improving patient prognosis.

## 1. Introduction

Autoimmune encephalitis (AE) is a complex neurological disorder triggered by an autoimmune response. First-line immunotherapy regimens include glucocorticoids, intravenous immunoglobulin (IVIg), and plasma exchange. Among these, glucocorticoids are the preferred initial treatment for AE due to their potent immunomodulatory effects and cost-effectiveness. However, long-term high-dose use often leads to severe side effects, while IVIg and plasma exchange carry potential risks and depend on limited blood supply resources.^[1]^ Therefore, there is an urgent need to develop new treatment strategies. Diencephalic lesions can manifest as various syndromes depending on the affected neural structures, including endocrine dysfunction, water-electrolyte imbalances, sexual and sleep disorders, and autonomic dysregulation. Currently, reports on diencephalitis are extremely rare. Our hospital recently admitted a case of immunotherapy-dependent autoimmune diencephalitis. The patient showed significant improvement after adjunctive treatment with efgartigimod, providing new insights into the treatment of autoimmune diencephalitis.

## 2. Case description

**Figure 1.**
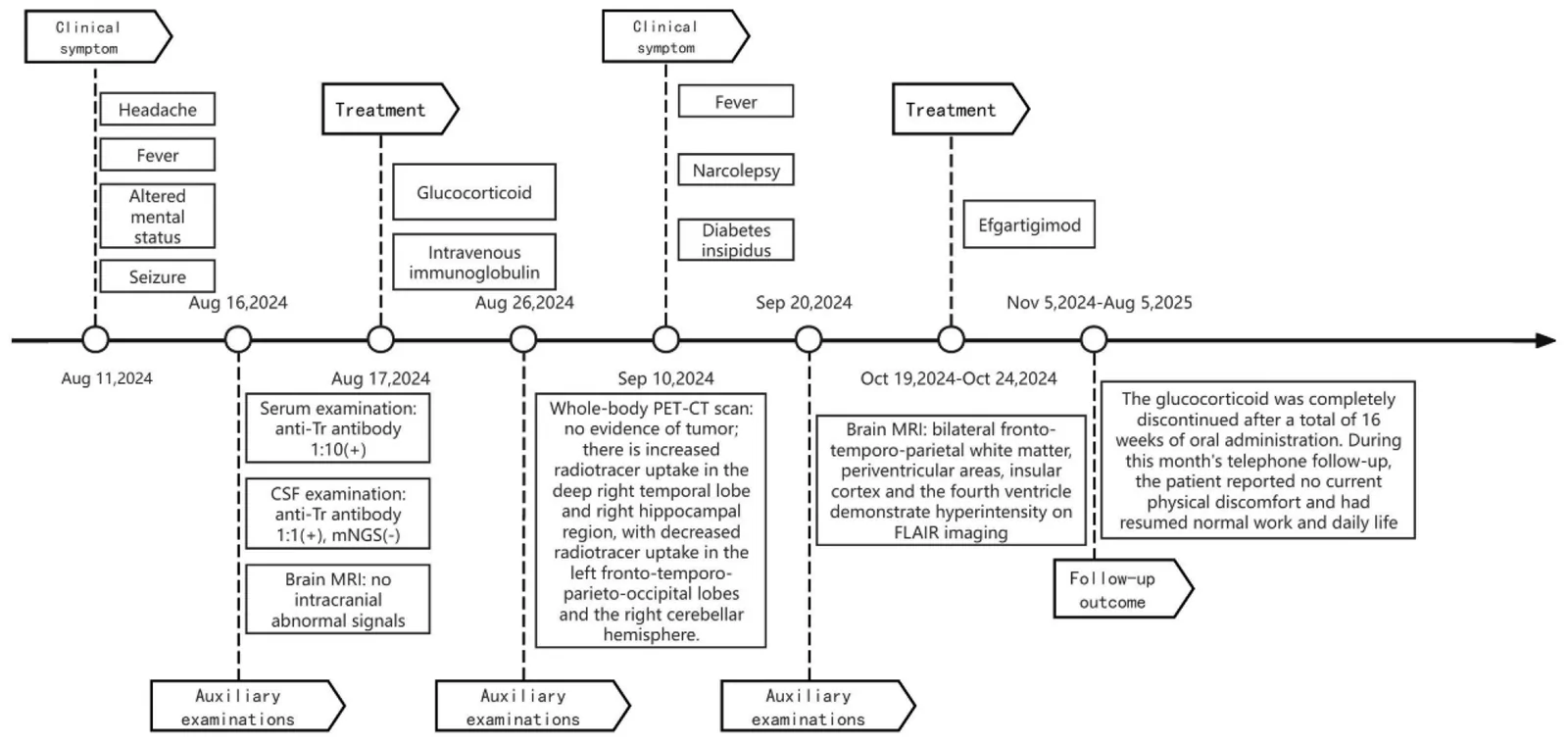
The timeline of the case. CSF = cerebrospinal fluid, mNGS = metagenomic next-generation sequencing, MRI = magnetic resonance imaging.

The timeline of the case (Fig. 1). On August 16, 2024, a 37-year-old male was admitted to our hospital due to “intermittent headache and fever for 5 days, worsened with seizures and impaired consciousness for 1 day.” The patient developed fever and headache 5 days prior, with a maximum body temperature of 39.7°C, and received anti-inflammatory treatment at a local clinic. One day ago, he experienced seizures and loss of consciousness, leading to his presentation at our hospital. Since the onset of symptoms, his sleep and diet have been poor, with normal bowel and bladder function and no significant recent weight changes. The patient was previously healthy and denied any relevant family medical history. Physical examination on admission showed body temperature 37°C, pulse 118 beats/min, respiratory rate 28 breaths/min, and blood pressure 145/78 mm Hg. The patient was in a light coma, with bilateral pupils equal and round, approximately 2.5 mm in diameter, direct and indirect light reflexes present, bilateral pathological signs negative, and the remaining neurological examination could not be cooperated with.

**Figure 2.**
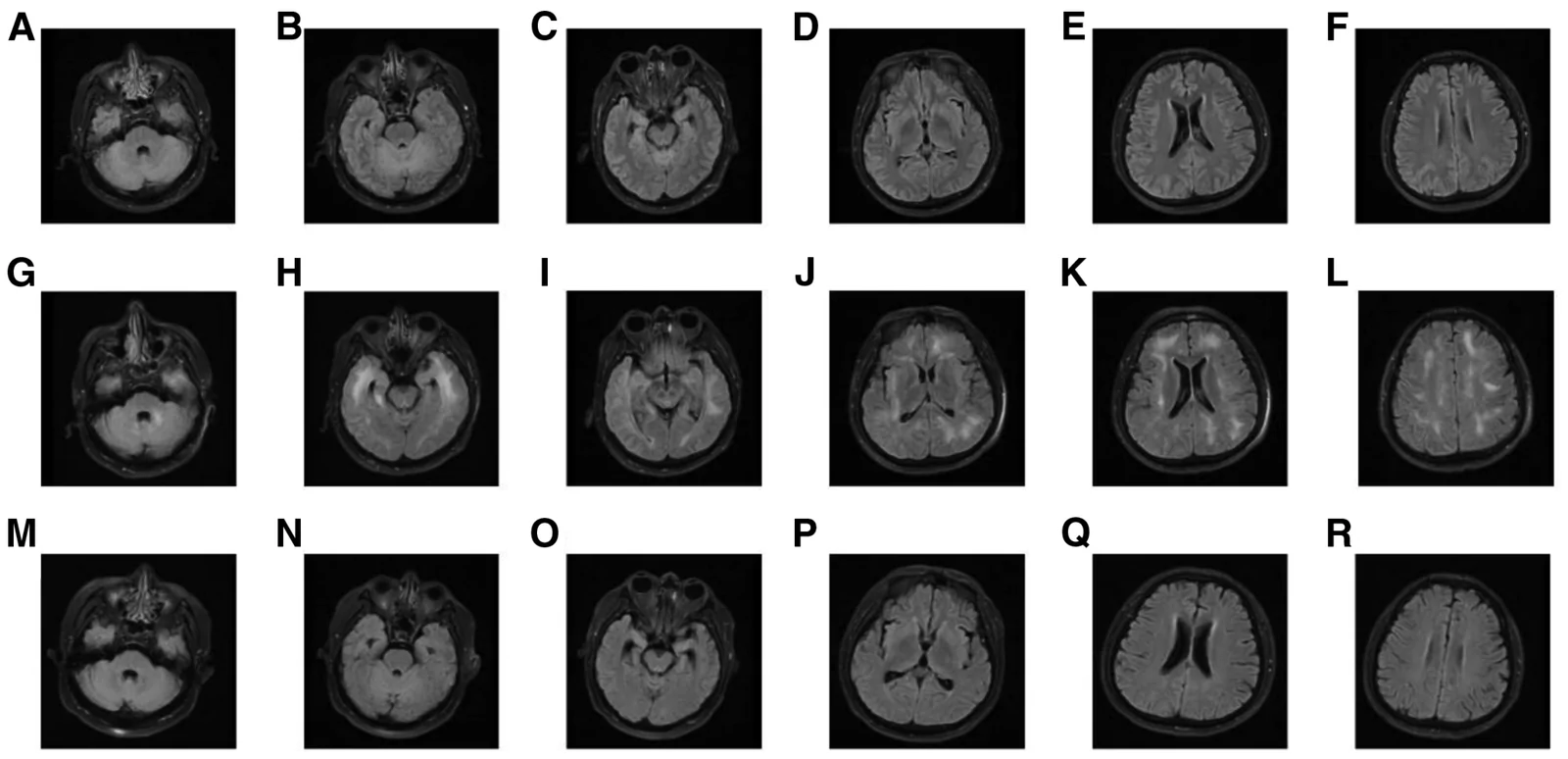
(A–F) Brain MRI showed no significant abnormal signals. (G–L) Brain MRI showed FLAIR hyperintensities in the bilateral fronto-temporo-parietal lobes, periventricular areas, insular white matter, and around the fourth ventricle. (M–R) Brain MRI showed no significant abnormal signals. FLAIR = fluid-attenuated inversion recovery, MRI = magnetic resonance imaging.

Brain magnetic resonance imaging (MRI) revealed no significant abnormal signals (Fig. 2A–F). Lumbar puncture (August 16) results showed cerebrospinal fluid (CSF) was colorless and clear, with an opening pressure >400 mmH_2_O; white blood cell count (WBC) 242 × 10^6^/L, protein 1.74 g/L, glucose 4.89 mmol/L, and chloride 113.9 mmol/L. Viral or bacterial encephalitis was highly suspected. The patient was treated with dehydration therapy for intracranial pressure reduction, antivirals, antibiotics, and supportive care. However, the patient continued to have persistent high fever, with a maximum temperature of 40°C.

**Figure 3.**
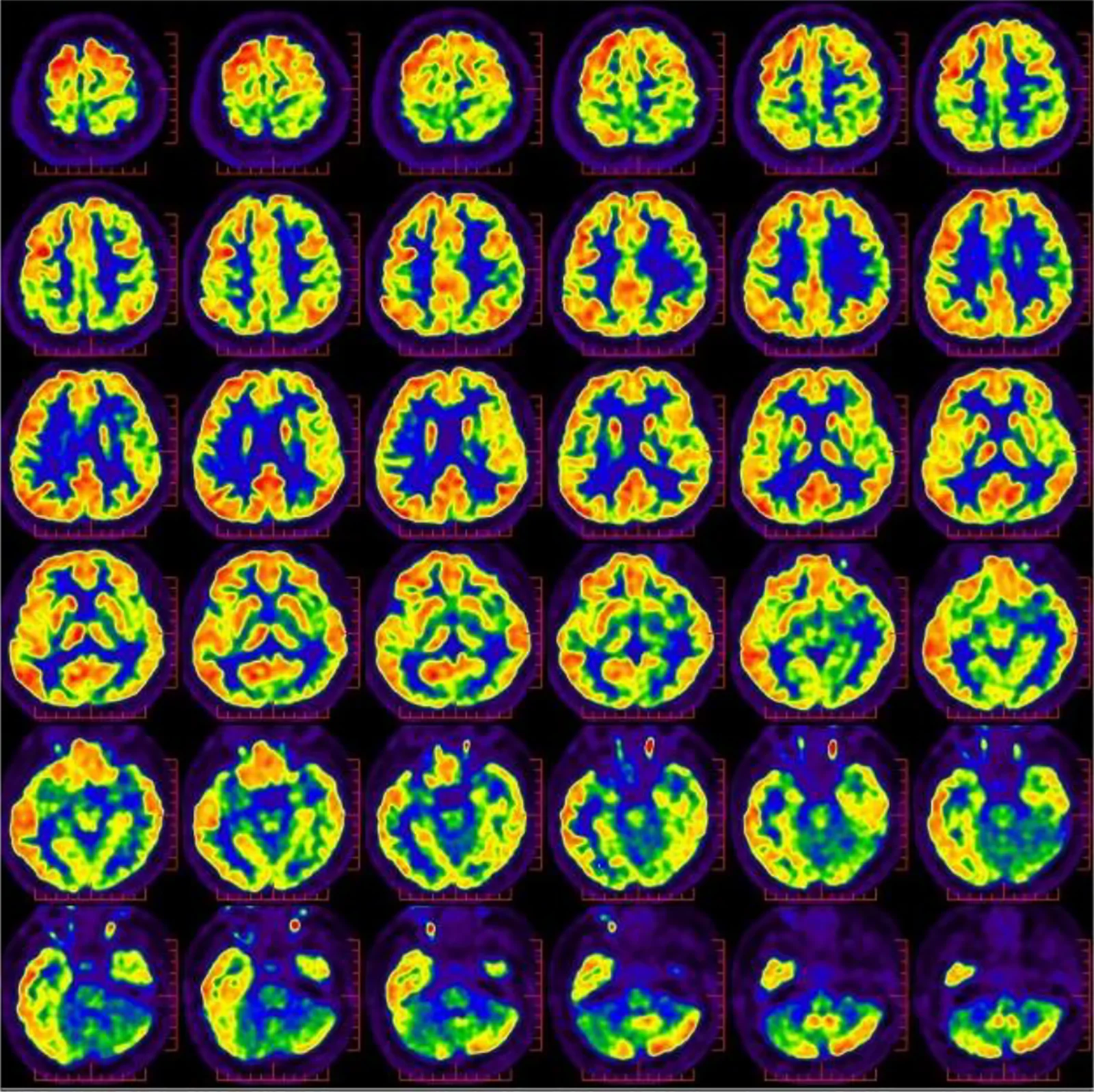
PET/CT showed increased radiotracer uptake in the deep right temporal lobe and right hippocampal region, and decreased uptake in the left fronto-temporo-parieto-occipital lobes and right cerebellar hemisphere. PET/CT = positron emission tomography/computed tomography.

**Figure 4.**
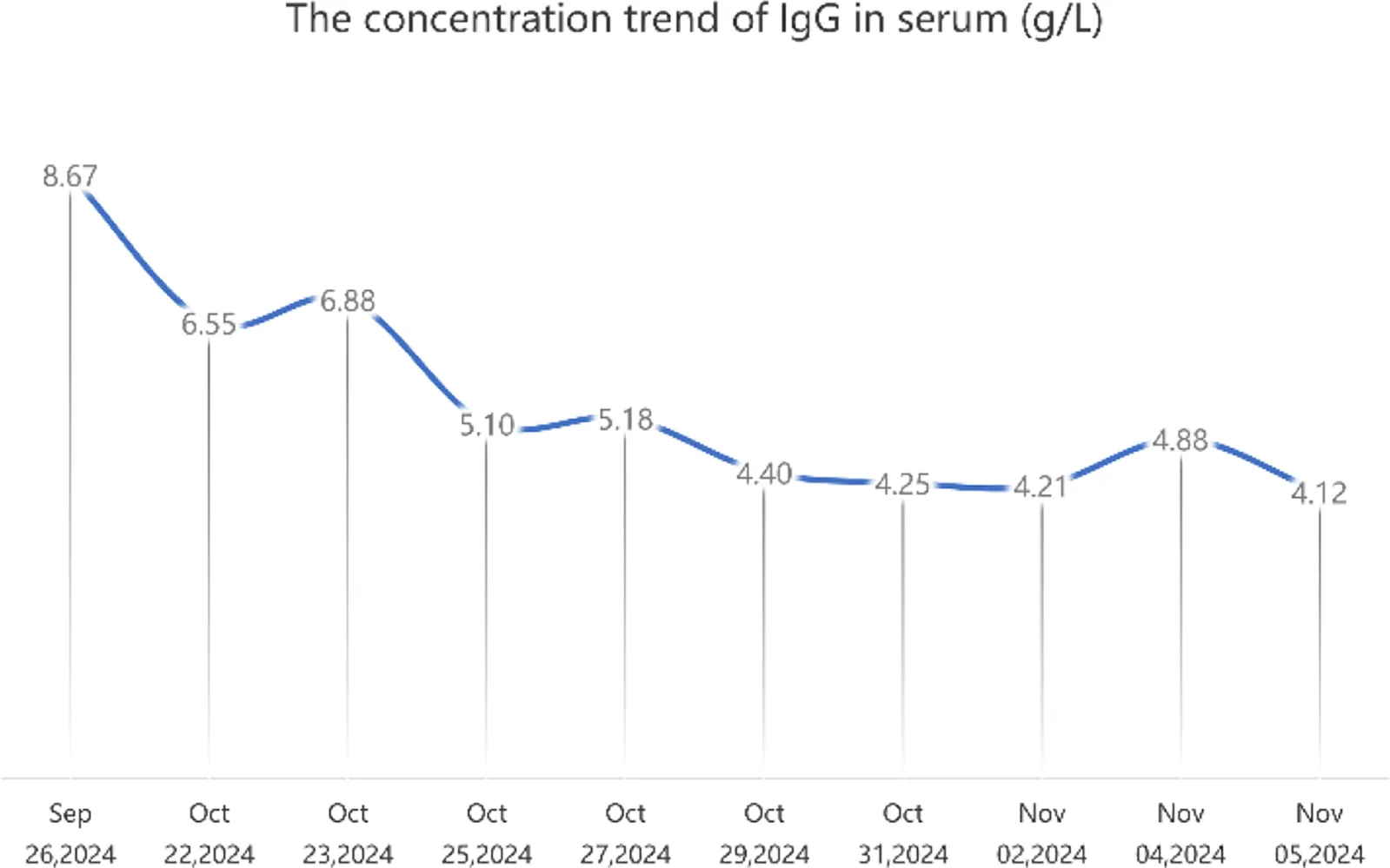
Changes in serum IgG concentration before and after efgartigimod treatment. IgG = immonoglobulin G.

AE antibody panel (tested by DIAN Diagnostics) results returned: anti-Tr antibody titer was 1:10 in serum and 1:1 in CSF. Pathogen metagenomic next-generation sequencing (mNGS) of the CSF detected no pathogens. Positron emission tomography-computed tomography (PET-CT) scan showed increased radiotracer uptake in the deep right temporal lobe and right hippocampal region, and decreased uptake in the left fronto-temporo-parieto-occipital lobes and right cerebellar hemisphere (Fig. 3). Electroencephalography showed irregular sharp waves and sharpened slow waves in the right temporal region during the interictal period. Antibody-mediated AE was considered. The patient received methylprednisolone pulse therapy with a tapering regimen (1000 mg on days 1–3, 500 mg on days 4–6, and 240 mg on days 7–9) and IVIg for immunomodulation for 5 days. The patient’s consciousness cleared, he became responsive and coherent, and his body temperature essentially normalized. However, when the methylprednisolone dose was reduced to 120 mg, the patient developed recurrent high fever and lethargy. A repeat lumbar puncture (August 27) showed CSF was colorless and clear, pressure >400 mmH_2_O; WBC count 464 × 10^6^/L, protein 1.76 g/L, glucose 3.19 mmol/L, chloride 113.8 mmol/L. Compared with the first lumbar puncture, the WBC count was higher, glucose was lower, while protein and chloride levels were largely unchanged. CSF mNGS again detected no pathogens. When methylprednisolone was reduced to 80 mg, the episodes of high fever (up to 40°C) and lethargy became significantly more frequent, characterized by high fever without sweating. Another lumbar puncture (September 5) showed CSF was colorless and clear, pressure 95 mmH_2_O; WBC count 205 × 10^6^/L, protein 0.58 g/L, glucose 4.26 mmol/L, chloride 125.9 mmol/L. CSF mNGS remained negative for pathogens. At this point, the patient developed diabetes insipidus (24-hour urine output > 6000 mL) and refractory hyponatremia. A third lumbar puncture (September 13) showed CSF was colorless and clear, pressure 355 mmH_2_O; WBC count 182 × 10^6^/L, protein 0.51 g/L, glucose 3.81 mmol/L, chloride 119.6 mmol/L. Repeat AE antibody panel (tested by KingMed Diagnostics) returned: anti-Tr antibody titer was 1:30 in serum and negative in CSF; CSF orexin A level was 89.78 pg/mL (≤110.00 pg/mL for Narcolepsy type 1). A diagnosis of autoimmune diencephalitis was considered, with relapse attributed to steroid tapering. The methylprednisolone dose was increased back to 240 mg intravenous. The patient’s temperature normalized, consciousness cleared gradually, and he became coherent again. These symptoms remained stable. Repeat brain MRI showed fluid-attenuated inversion recovery hyperintensities in the bilateral fronto-temporo-parietal lobes, periventricular areas, insular white matter, and around the fourth ventricle (Fig. 2G–L). A fourth lumbar puncture (September 27) showed CSF was colorless and clear, pressure 170 mmH_2_O; WBC count 24 × 10^6^/L, protein 0.40 g/L, glucose 3.37 mmol/L, chloride 120.8 mmol/L. Another attempt to reduce methylprednisolone to 120 mg led to recurrence of high fever and episodic lethargy. Subsequently, a more gradual taper was attempted (240 mg → 220 mg → 200 mg → 160 mg) supplemented with mycophenolate mofetil (MMF). The episodic lethargy improved, but the patient still had intermittent fever (up to 38.5°C), indicating difficulty in steroid tapering. The patient then received efgartigimod alfa injection for antibody clearance (October 19 and October 24). Serum immonoglobulin G (IgG) levels were monitored dynamically (Fig. 4). The patient’s high fever and episodic lethargy improved significantly, almost returning to normal. Repeat brain MRI showed no significant abnormal signals (Fig. 2M–R). After discharge, the patient followed a protocol to gradually taper oral glucocorticoids, starting from 80 mg and reducing by 5 mg weekly, completely discontinuing them after 16 weeks. In a telephone follow-up this month, the patient reported no discomfort and had fully resumed normal work and life.

## 3. Discussion

The diagnostic criteria for diencephalitis include excessive daytime sleepiness, narcolepsy-cataplexy, low CSF orexin levels, hyperthermia, hypothalamic-pituitary endocrine dysfunction, and weight gain or sexual dysfunction.^[2]^ Previous reports have associated diencephalitis with anti-Ma2 antibodies, typically linked to testicular cancer and non-small cell lung cancer.^[3]^ In this case, 2 separate antibody tests (conducted at different laboratories) returned positive for anti-Tr antibodies but negative for anti-Ma2 antibodies. A whole-body PET-CT scan ruled out malignancy. The patient exhibited excessive daytime sleepiness, hyperthermia, hyperphagia, weight gain, and diabetes insipidus, along with diencephalic abnormalities on MRI, confirming the diagnosis of diencephalitis.

Delta/Notch-like epidermal growth factor–related receptor (DNER) is expressed by Purkinje cells and plays a crucial role in neuron-glia signaling.^[4]^ Anti-Tr antibodies were first discovered in 1997, and in 2012, their target antigen was identified as the extracellular domain of DNER.^[5]^ Anti-Tr antibodies are autoantibodies associated with paraneoplastic neurological syndromes and are strongly linked to malignancies, particularly Hodgkin lymphoma–associated cerebellar degeneration, which shows high specificity. There have also been reports of associations with non-Hodgkin lymphoma, lung cancer, and tongue cancer.^[6,7]^ Patients with anti-Tr antibody positivity typically present with cerebellar ataxia, while extracerebellar manifestations are rare.^[7]^ These may include cognitive impairment, psychomotor slowing, reduced verbal fluency, impaired mental flexibility, and seizures. In addition, peripheral nervous system symptoms may occur, such as pain, paresthesia, hypesthesia, sensory ataxia, fasciculations, and muscle atrophy.^[8]^ This is closely related to the fact that DNER is almost exclusively expressed in the cerebellum in humans.^[9]^ A 2003 study by Bernal et al reported 28 anti-Tr-positive ataxia cases, of which 25 had pure cerebellar involvement, 2 exhibited encephalopathy and sensory neuropathy, and only 1 presented as limbic encephalitis.^[10]^

On one hand, this patient tested positive for anti-Tr antibodies. According to paraneoplastic syndrome consensus guidelines, anti-Tr antibodies are classified as high-risk paraneoplastic antibodies strongly suggestive of Hodgkin lymphoma. Typically, these antibodies manifest clinically as rapidly progressive cerebellar ataxia, which was inconsistent with this patient’s presentation. Furthermore, whole-body PET-CT imaging failed to reveal any evidence of malignancy. On the other hand, the patient presented with severe encephalitis, and anti-Tr antibodies were detected in both CSF and serum. Empirical anti-infective therapy proved ineffective, whereas treatment with glucocorticoids, IVIg, MMF, and efgartigimod yielded significant clinical improvement. However, there are currently no reported cases of anti-Tr antibody-mediated encephalitis manifesting with such clinical features. Thus, it remains unclear whether the anti-Tr antibodies in this patient played a pathogenic role or merely served as “bystanders” in the disease process.

Efgartigimod is a humanized IgG1 antibody fragment engineered using ABDEG technology that competitively binds to the neonatal Fc receptor (FcRn), blocking its interaction with IgG and thereby inhibiting IgG recycling while promoting the degradation of both IgG and pathogenic autoantibodies.^[11]^ As the world’s first FcRn antagonist, multiple clinical trials in recent years have demonstrated its efficacy in significantly improving clinical symptoms in patients with myasthenia gravis (MG), leading to its approval for MG treatment in the United States, Europe, Japan, and China.^[12,13]^ Beyond MG, efgartigimod has shown therapeutic benefits for various antibody-mediated autoimmune diseases, including immune thrombocytopenia, pemphigus vulgaris, and pemphigus foliaceus.^[14]^ While its mechanism of action in central nervous system disorders remains incompletely understood, potential pathways may include peripheral antibody clearance effect, blood–brain barrier penetration hypothesis, and local FcRn inhibition.^[15]^ In the present case of refractory autoimmune diencephalitis, we administered efgartigimod twice according to the prescribing information (in the absence of specific treatment guidelines), achieving favorable therapeutic outcomes before discontinuing efgartigimod and maintaining treatment with glucocorticoids and MMF.

## 4. Limitations

This report has several limitations: the observed therapeutic effects cannot be attributed solely to efgartigimod monotherapy, as synergistic effects from glucocorticoids, IVIg, and MMF must be considered; being a single-center case report, individual variability may influence outcomes; and optimal dosing regimen parameters (including dosage and administration frequency) for efgartigimod remain undetermined.

This case demonstrates the favorable effect of efgartigimod as an adjunctive therapy for autoimmune diencephalitis, effectively controlling symptoms and improving patient prognosis. It provides valuable clinical insights for the treatment of refractory autoimmune diencephalitis.

## Acknowledgments

We thank all members of the research team, as well as the patient and his families.

## Author contributions

**Conceptualization:** Xiang Yin.

**Investigation:** Menghan Jia.

**Data curation:** Jie Shao.

**Writing – review & editing:** Li Cui.

**Writing – original draft:** Chang Li.
